# Identification of a New Cyclovirus in Cerebrospinal Fluid of Patients with Acute Central Nervous System Infections

**DOI:** 10.1128/mBio.00231-13

**Published:** 2013-06-18

**Authors:** Le Van Tan, H. Rogier van Doorn, Ho Dang Trung Nghia, Tran Thi Hong Chau, Le Thi Phuong Tu, Michel de Vries, Marta Canuti, Martin Deijs, Maarten F. Jebbink, Stephen Baker, Juliet E. Bryant, Nguyen Thi Tham, Nguyen Thi Thuy Chinh BKrong, Maciej F. Boni, Tran Quoc Loi, Le Thi Phuong, Joost T. P. Verhoeven, Martin Crusat, Rienk E. Jeeninga, Constance Schultsz, Nguyen Van Vinh Chau, Tran Tinh Hien, Lia van der Hoek, Jeremy Farrar, Menno D. de Jong

**Affiliations:** Oxford University Clinical Research Unit, Wellcome Trust Major Overseas Programme, South East Asia Infectious Diseases Clinical Research Network, Hospital for Tropical Diseases, Ho Chi Minh City, Vietnam^a^;; Centre for Tropical Medicine, Nuffield Department of Clinical Medicine, University of Oxford, Centre for Clinical Vaccinology and Tropical Medicine, Oxford, United Kingdom^b^;; Pham Ngoc Thach University of Medicine, Ho Chi Minh City, Vietnam^c^;; Hospital for Tropical Diseases, Ho Chi Minh City, Vietnam^d^;; Department of Medical Microbiology, Academic Medical Center, University of Amsterdam, Amsterdam, the Netherlands^e^;; Dong Thap Provincial Hospital, Dong Thap, Vietnam^f^;; Department of Global Health-Amsterdam Institute of Global Health and Development, Academic Medical Center, University of Amsterdam, Amsterdam, The Netherlands^h^

## Abstract

Acute central nervous system (CNS) infections cause substantial morbidity and mortality, but the etiology remains unknown in a large proportion of cases. We identified and characterized the full genome of a novel cyclovirus (tentatively named cyclovirus-Vietnam [CyCV-VN]) in cerebrospinal fluid (CSF) specimens of two Vietnamese patients with CNS infections of unknown etiology. CyCV-VN was subsequently detected in 4% of 642 CSF specimens from Vietnamese patients with suspected CNS infections and none of 122 CSFs from patients with noninfectious neurological disorders. Detection rates were similar in patients with CNS infections of unknown etiology and those in whom other pathogens were detected. A similar detection rate in feces from healthy children suggested food-borne or orofecal transmission routes, while high detection rates in feces from pigs and poultry (average, 58%) suggested the existence of animal reservoirs for such transmission. Further research is needed to address the epidemiology and pathogenicity of this novel, potentially zoonotic virus.

## IMPORTANCE

Acute central nervous system (CNS) infections cause substantial morbidity and mortality, but the etiology frequently remains unknown, which hampers development of therapeutic or preventive strategies. Hence, identification of novel pathogens is essential and is facilitated by current next-generation sequencing-based methods. Using such technology, we identified and characterized the full genome of a novel cyclovirus in cerebrospinal fluid (CSF) specimens from two Vietnamese patients with CNS infections of unknown etiology, which was subsequently detected in none of 122 CSF specimens from patients with noninfectious neurological disorders but 4% of 642 CSF specimens from Vietnamese patients with suspected or confirmed CNS infections. Similar detection rates in feces from healthy children suggested food-borne or orofecal transmission routes, while frequent detection in feces from Vietnamese pigs and poultry (average, 58%) suggested the existence of animal reservoirs for such transmission. Further studies are needed to address the epidemiology and pathogenicity of this novel, potentially zoonotic virus.

## Introduction

Acute central nervous system (CNS) infections cause substantial morbidity and mortality in children and adults, especially in tropical regions. While the causative microorganisms include a wide variety of bacterial, parasitic, fungal, and particularly viral pathogens, the majority of cases remain undiagnosed, despite extensive diagnostic efforts (1). The identification of previously unknown pathogens in patients with acute CNS infections remains essential to improve prevention and clinical management of this frequently devastating syndrome. The discovery of such novel pathogens is facilitated by the availability of sensitive sequence-independent molecular methods.

Using a combination of an amplified fragment length polymorphism (AFLP)-based virus discovery assay (VIDISCA) and next-generation sequencing (2), we set out to look for nucleic acid sequences of potential novel pathogens in cerebrospinal fluid (CSF) specimens from Vietnamese adults and children with acute CNS infections of unknown etiology. Here we report the identification and full genome characterization of a novel virus belonging to the recently proposed genus *Cyclovirus* in the family *Circoviridae*. In addition, we report the results of initial studies to explore the pathogenic potential of this virus in humans as well as the existence of possible animal reservoirs for zoonotic transmission.

## RESULTS

### Identification of a cyclovirus-like sequence in cerebrospinal fluid.

**FIG 1:**
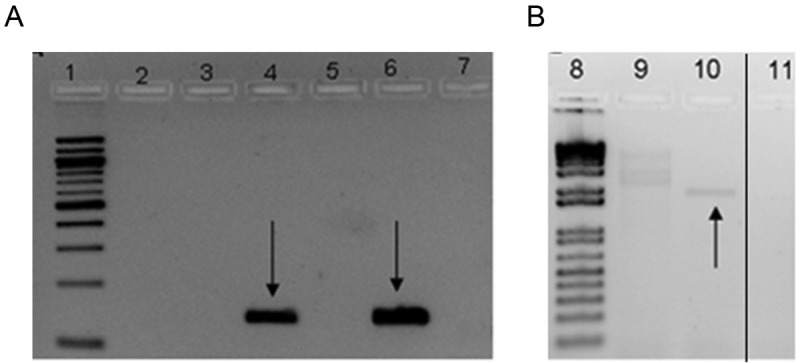
Gel electrophoresis of PCR products. (A) Cyclovirus-like sequence PCR; (B) inverse PCR of cyclovirus. Lanes 1 and 8, 100-bp and 1-kb ladders, respectively; lanes 2 to 6, 9, and 10, patient samples; lanes 7 and 11, negative controls. Arrows indicate products of expected sizes. The thin black line indicates the spliced margin where the two unrelated lanes between lanes 10 and 11 were removed from the original gel picture.

The analysis of the (pooled) CSF specimens generated 321,000 reads, which were reduced to 161,000 sequence reads for further analysis after excluding *Escherichia coli-*like sequences suspected to be derived from VIDISCA-454 (454: Roche 454 Genome Sequencer FLX System) reagents (data not shown). Of these, a 190-bp sequence was identified in a pool consisting of 5 CSF samples that showed 82% identity to a replication-associated gene (*Rep*) of cycloviruses (CyCV). To confirm this VIDISCA-454 read, a specific PCR (Table 1) targeting the sequence was employed and successfully detected a fragment of the expected size in 2 out of 5 original CSF samples (Fig. 1A). The fragment was sequenced, which confirmed the CyCV-like sequence that was obtained from the VIDISCA-454 analysis (data not shown). These results indicated that a CyCV-like virus was present in CSF of two patients (one child and one adult) (Table 2) with acute CNS infections of unknown etiology.

**TABLE 1:** Oligonucleotide sequences of primers and a probe used in this study

Primers	Oligonucleotide sequence (5′–3′)	Source
CyCV31_53F	GAGCGCACATTGAAAGAGCTAAA	Newly designed
CyCV178-153R	TCTCCTCCTTCAATGACAGAAACAAC	Newly designed
CyCV65-96Probe*	FAM-CGADAATAAGGMATACTGCTCTAAAGSTGGCG-BHQ1	Newly designed
CVF1	GGIAYICCICAYYTICARGG	3
CVR1	AWCCAICCRTARAARTCRTC	3
CVF2	GGIAYICCI CAYYTICARGGITT	3
CVR2	TGYTGYTCRTAICCRTCCCACCA	3
CyCV31_53F-rc	TTTAGCTCTTTCAATGTGCGCTC	Newly designed
CyCV178-153R-rc	GTTGTTTCTGTCATTGAAGGAGGAGA	Newly designed
CyCV_FL1	ACTTATTTCTAATTCATATTGCCGGGTA	Newly designed
CyCV_FL2	TCAGCGTCCAGCAGAATCTAC	Newly designed
CyCV_FL3	AAGCTCATCGTATTTGATCCATCC	Newly designed
CyCV_FL4	CCTCACTGAGCTATGTAAATTTGCT	Newly designed

**TABLE 2:** Characteristics and clinical outcome of patients with CNS infections and positive for CyCV-VN by PCR

Group and patient (*n*)^*a*^	Other confirmed diagnosis^*b*^	Sample date	No. (%) of males in group or sex of patient^*c*,*d*^	Age (yr)^*d*^	Length of hospital stay (days)^*d*^	Illness day	No. of patients with^*d*^:							CSF white blood cell count	% of lympho- cytes in CSF	CSF/blood glucose ratio	Out- come^*f*^	Sequelae at discharge
							Fever	Convul- sions	Neck stiff- ness	Limb weak- ness	Head- ache	Vom- iting	GCS score^*e*^					
Group 1 (12)			9 (75)	19.5 (<1–50)	8 (2–41)	5 (2–20)	11 (92)	4 (33)	7 (58)	2 (18)	6 (86)	8 (89)		40 (1–464)				
1	None	Jan. 5, 2004	F	<1	7	5	Y	N	N	Y	NA	NA	2 (BCS)	1		0.88	1	None
2	None	Jun. 8, 2004	M	<1	8	7	Y	N	N	N	NA	NA	3 (BCS)	3		0.53	1	None
3	None	Feb. 7, 2004	M	12	8	3	Y	N	N	N	NA	NA	6	26	90	0.96	1	None
4	None	Oct. 18, 1999	M	27	5	7	Y	N	Y	NA	NA	Y	15	54	68	0.55	1	None
5	None	Jun. 12, 2008	M	31	2	2	Y	Y	N	N	Y	Y	15	10		0.54	3	Unknown
6	None	Nov. 25, 2009	F	<2	9	2	Y	Y	N	N	NA	Y	5 (BCS)	10	60	0.56	1	Unknown
7	None	Nov. 29, 2008	M	20	41	20	Y	Y	Y	Y	Y	Y	7	160	30	0.73	1	Unknown
8	None	Sep. 4, 2009	M	15	15	9	Y	N	Y	N	Y	Y	15	78	20	0.62	1	Unknown
9	None	Sep. 7, 2009	F	50	15	7	Y	N	Y	N	Y	Y	15	105	22	0.44	1	Unknown
10	None	Nov. 18, 2009	M	19	5	5	Y	Y	Y	N	Y	N	11	14	36	0.55	1	Unknown
11	None	Mar. 31, 2008	M	43	3	5	N	N	Y	N	Y	Y	9	78	20	0.57	3	Unknown
12	None	Apr. 16, 2008	M	24	17	4	Y	N	Y	N	N	Y	15	464	20	0.51	1	Unknown

Group 2 (4)			3 (75)	14 (2–19)	14 (4–38)	5.5 (2–7)	2 (50)	3 (100)	2 (50)	1 (25)	2 (100)	2 (100)		50 (0–110)				
1	JEV	Jan. 30 2004	M	2	4	2	N	N	N	N	NA	NA	13	0		0.98	1	None
2	JEV	Aug. 12 2004	M	11	5	5	Y	N	Y	N	NA	NA	11	22	65	0.81	1	None
3	DENV	Oct. 24 2008	M	19	38	7	N	NA	Y	Y	Y	Y	9	79	21	0.41	1	Unknown
4	JEV	May 05 2008	F	17	23	6	Y	N	N	N	Y	Y	15	110	25	0.71	1	Unknown

Group 3 (7)			6 (85)	5 (<1–61)	19 (1–34)	2 (1–3)	6 (85)	2 (28)	5 (71)	1 (14)	4 (100)	4 (57)		1,439 (2–9,160)				
1	*S. suis*	Aug. 31, 2008	M	29	21	1	N	N	Y	N	Y	Y	15	3,500		0.04	1	Unknown
2	*S. pneumoniae*	Nov. 17, 2008	M	25	16	2	Y	N	Y	N	Y	Y	15	5,513	20	0.23	1	Unknown
3	*H. influenzae*	Jan. 20, 2009	M	<1	19	1	Y	N	Y	N	NA	N	5 (BCS)	9,160	9	0.36	1	Unknown
4	*S. pneumoniae*	Jan. 27, 2009	M	5	1	1	Y	Y	N	Y	Y	Y	8	30	11	0.18	2	
5	*S. suis*	Mar. 16, 2009	F	61	34	3	Y	N	Y	N	NA	N	11	1,439	28	0.19	1	Unknown
6	*S. pneumoniae*	Apr. 17, 2009	M	2	4	2	Y	Y	N	N	NA	N	0 (BCS)	2		0.65	1	Unknown
7	*S. pneumoniae*	Dec. 23, 2009	M	3	20	2	Y	N	Y	N	y	Y	11	80	33	0.95	1	Unknown

Group 4 (5)			1 (20)	32 (29–68)	4 (2–10)	5 (3–7)	4 (80)	0	4 (80)	0	NA	NA		296 (100–950)				
1187	*M. tuberculosis*	Aug. 4, 2009	F	30	4	7	Y	N	Y	N	NA	NA	13	296	NA	0.18	3	Unknown
1847	None	Feb. 16, 2009	M	32	10	3	Y	N	Y	N	NA	NA	15	100	20	0.13	1	Unknown
1854	None	Jul. 24, 2009	F	29	2	3	Y	N	N	N	NA	NA	15	603	20	0.34	1	Unknown
2117	*M. tuberculosis*	Feb. 9, 2010	F	55	NA	7	N	Unknown	Y	N	NA	NA	7	950	60	NA	3	Unknown
2460	None	Nov. 13, 2008	F	68	NA	5	Y	N	Y	N	NA	NA	15	296	NA	0.72	3	Unknown

^a^ Group 1, patients with CNS infections of unknown etiology; group 2, patients with laboratory-confirmed CNS infections; group 3, bacterial meningitis patients; group 4, PCR confirmed/clinically suspected tuberculosis meningitis patients. Names of provinces from which the patients were originated are intentionally not shown; patients 5 and 6 were the index cases in whose samples the CyCV-like sequence was detected by VIDISCA-454.

^b^ For *M. tuberculosis*, denominators may vary. JEV, Japanese encephalitis virus; DENV, dengue virus.

^c^ M, male; F, female.

^d^ Data are numbers (percentages) of patients; continuous variables are presented as medians (ranges). NA, not available; Y, yes; N, no.

^e^ Scores are on the Glasgow coma scale (GCS) unless the Blantyre coma scale (BCS) is indicated.

^f^ 1, recovery; 2, death; 3, unknown.

### Genome characterization and phylogenetic analysis of a new cyclovirus.

**FIG 2:**
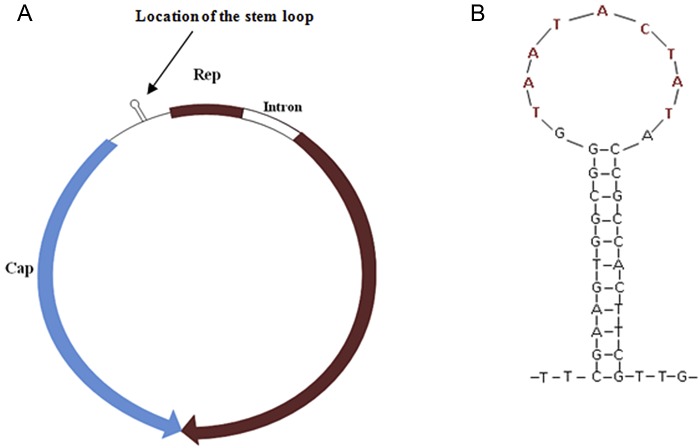
(A)Predicted genome organization of CyCV-VN; (B) stem-loop structure of CyCV-VN, with nonamer sequence in red.

To further characterize the CyCV-like virus, an initial attempt was made to amplify the partial Rep gene (primers CVFs and CVRs) (Table 1), but this was unsuccessful. Therefore, inverse PCR (iPCR) was used for direct amplification of the viral genome from clinical specimens. An amplified product of less than 2 kb was successfully obtained from only one sample (Fig. 1B) and was then subjected to direct sequencing with use of iPCR primers and primers FL1 to FL4 (Table 1) to generate the full viral genome. The cyclovirus species of the completed genome was tentatively named cyclovirus-Vietnam (CyCV-VN). The single-stranded circular DNA genome consisted of 1,856 bp and revealed a genomic structure characteristic of the genera *Circovirus* and *Cyclovirus* in the family *Circoviridae* with the typical feature of two open reading frames (ORFs) encoding two major proteins, the replication-associated (Rep) and capsid (Cap) proteins, arranged in opposite directions (Fig. 2A). These two deduced proteins are 292 amino acids (aa) and 222 aa long, respectively. The Rep gene of CyCV-VN was predicted to be interrupted by a 166-bp intron with a typical splice donor (GT) and splice acceptor site (AG), as in other CyCVs (3, 4). Amino acid sequencing of the Rep protein showed that the conserved motifs (WWDGY, DDFYGW, DRYP, FTLNN, TPHLQG, CSK, and G-GSK) that are commonly observed among circoviruses, including CyCVs, were also found in CyCV-VN (data not shown) (3). As in other CyCVs, the intergenic region between the 3′ ends of the two ORFs was absent (3–5), and a highly conserved stem-loop structure was present at the 5′ intergenic region of the viral genome (Fig. 2B). The latter structure is thought to initiate the rolling-cycle replication of the virus (6). The nonamer sequence of CyCV-VN (TAATACTAT) was identical to that of other CyCVs (Table 3), and the sequence of the loop was 11 bp long, which is within the 9- to 13-bp range of other CyCV loops (3–5) (Table 3).

**TABLE 3:** Nonamer sequences of CyCV-VN and 15 different CyCV species and lengths of the stems

CyCV species	Nonamer sequence^*a*^	Loop length
VN	TAATACTAT	11
PK 5006	TAATACTAT	13
PK 5034	TAATACTAT	13
PK 5222	TAATACTAT	13
PK 5510	TAATACTAT	12
Chimp 11	TAATACTAT	13
NG12	TAATACTAT	13
NG 14	TAATACTAT	11
TN 18	TAATACTAT	12
NG chicken 8	TAATACTAA	12
PK goat 11	TAATACTAT	12
PK goat 21	TAATACTAG	13
TB bat USA	TAATACTAT	12
Bat GF-4c	TAATACTAT	11
NG 13	TAGTATTAC	9
DfCyCV-A1	TAATACTAT	13
FWCasCyV-GS140	TAGTATTAC	11

^a^ Underlined nucleotides are those that differ from the consensus sequence.

**FIG 3:**
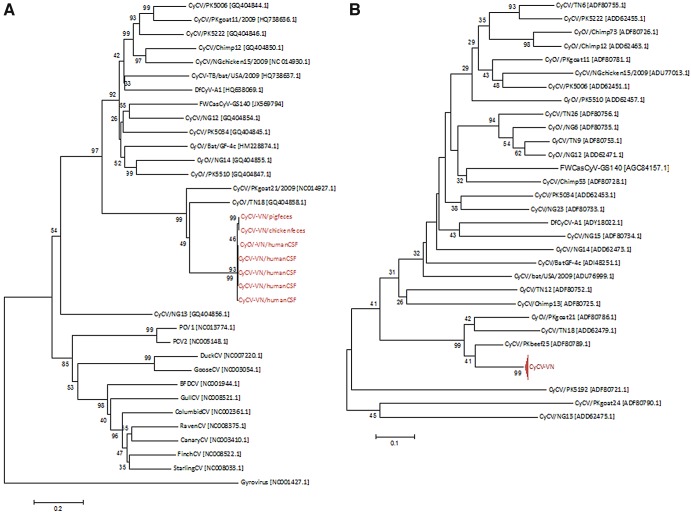
Reconstructed phylogeny trees of cycloviruses. (A) Tree based on complete genome sequences of 7 strains of CyCV-VN (red), 16 cycloviruses, 12 circoviruses, and a gyrovirus as an outlier. (B) Tree based on partial amino acid sequences of Rep proteins of 28 reported CyCV species and 27 CyCV-VN strains, indicated by the compressed branch. Trees were built by means of neighbor joining (MEGA 4.1); bootstrap tests of the reconstructed trees were done with 1,000 replicates. Sequence accession numbers are in brackets. CV, circovirus.

There are no formally defined criteria for classifying a new viral species within the recently proposed genus *Cyclovirus*. However, criteria for a new species within the genus *Circovirus* proposed by the International Committee on Taxonomy of Viruses are whole-genome sequence identities of less than 75% at the nucleotide level and amino acid sequence identities of the Cap protein of less than 70% (6). Li and colleagues, who first described the genus *Cyclovirus*, proposed to use these species demarcation criteria of the genus *Circovirus* to classify cycloviral species (3). To determine whether CyCV-VN represents a new cyclovirus species, its complete genome sequence was compared to 15 representative full-length genomes of 15 different cyclovirus species. The degrees of similarity ranged from 44 to 70% at the nucleotide level, with highest similarity being observed with CyCV-TN18, a cyclovirus detected in the stool of a Tunisian patient with acute flaccid paralysis (Table 4). Phylogenetic analysis confirmed that CyCV-VN was genetically distinct from known cycloviruses, albeit most closely related to CyCV-TN18 and CyCV-TN25, both derived from human stool samples from Tunisia and belonging to the same species as proposed by Li et al. (Fig. 3A) (3). Analysis of amino acid sequences of the Cap proteins from the same viruses yielded results similar to those of whole-genome analysis (Table 4).

**TABLE 4:** Degree of sequence identities between CyCV-VN and other cycloviruses^*a*^

CyCV species	% identity of:			Host
	Complete genome (nt level)	Cap protein (aa level)	Rep protein (aa level)	
USAbat-TB/2009	46	22	51	Bats
BatGF-4c	45	22	50	Bats
Chimp12	43	24	46	Chimpanzees
NG12	44	26	51	Humans
NG13	40	14	43	Humans
NG14	44	22	50	Humans
NGchicken15/2009	45	23	49	Chickens
PK5006	46	22	48	Humans
PK5034	44	23	51	Humans
PK5222	44	30	49	Humans
PK5510	46	28	51	Humans
PKgoat11/2009	44	25	49	Sheep
PKgoat21/2009	67	50	70	Sheep
TN18	70	48	74	Humans
DfCyCV-A1	50	30	47	Dragonflies
FWCasCyV-GS140	45	23	33	Cockroaches
NG23	NA	NA	50	Humans
Chimp13	NA	NA	51	Chimpanzees
PK5192	NA	NA	40	Humans
PKgoat24	NA	NA	30	Sheep
Pkbeef25	NA	NA	76	Bovines
TN12	NA	NA	46	Humans
TN9	NA	NA	46	Humans
TN26	NA	NA	49	Humans
NG6	NA	NA	46	Humans
NG15	NA	NA	46	Humans
Chimp73	NA	NA	45	Chimpanzees
TN6	NA	NA	46	Humans
Chimp53	NA	NA	48	Chimpanzees

^a^ NA, not available. Sequence accession numbers are shown in Fig. 3.

Twenty-nine cyclovirus species have been proposed thus far (3–5, 7). While complete genomes of only 15 species are available, partial sequences of the Rep gene are available for the remaining 13 species. It has recently been proposed that separate CyCV species should have less than 85% similarity at the amino acid level in the partial Rep protein (3, 4). To further determine the relationships between CyCV-VN and reported CyCVs for which complete genomes were not available, pairwise comparison and phylogenetic analysis were performed on nonrepetitive amino acid sequences of Rep proteins of 28 cyclovirus species. Results of these analyses confirmed the findings of our analyses of whole genome sequences and Cap proteins (Fig. 3B; Table 4).

### Prevalence of CyCV-VN DNA detection in CSF of patients with central nervous system disorders of suspected infectious and noninfectious etiology.

To study the prevalence of CyCV-VN in CSF, we first determined the frequency of viral DNA detection by CyCV-VN-specific real-time PCR in 642 randomly selected CSF samples from patients with suspected acute CNS infections enrolled in three descriptive studies in southern and central Vietnam from which the acute-infection CSF samples were selected for our virus detection experiments. We detected CyCV-VN in 26 of 642 (4%) acute-infection CSF specimens, including 10 of 273 (3.7%) CSF specimens from patients with CNS infections of unknown etiology and 16 of 369 (4.3%) from patients in whom laboratory-confirmed CNS infection with other pathogens was established. The latter included 4/140 (2.9%) patients with confirmed viral encephalitis (Japanese encephalitis virus [JEV] and Dengue virus [DENV]), 7/108 (6.5%) patients with bacterial meningitis (*Streptococcus pneumoniae*, *Haemophilus influenzae*, or *Streptococcus suis* serotype 2) and 5/121 (4.1%) patients with laboratory-confirmed or clinically suspected tuberculous meningitis (Table 2). The viral burden as estimated by cycle threshold (*C*_*T*_) values was not different between specimens of unknown etiology and those diagnosed with other pathogens (data not shown). Details on characteristics and clinical outcome of CyCV-VN patients are presented in Table 2 and are compatible with that of other patients with CNS infections, although the mortality observed (4%) was lower than the overall mortalities of patients in cohorts from the PVE (a pediatric viral encephalitis study), BMD (an adult viral encephalitis study), and 01SS (a study of children and adults with suspected CNS infections) studies (29%, 10% and 12%, respectively). CyCV-DNA could not be detected in 122 CSF specimens collected from Vietnamese patients with noninfectious neurological disorders.

### CyCV-VN detection in human specimens other than CSF.

We tested the presence of CyCV-VN DNA in available specimens other than CSF that were collected in parallel from patients whose CSF was positive and found CyCV-VN DNA in 1 of 5 rectal swabs, 1 of 4 throat swabs, and 0 of 3 available sera obtained at the time of lumbar puncture.

No viral DNA was detected in 90 residual blood specimens obtained from routine hospital biochemistry laboratories in Vietnam. However, CyCV-VN DNA could be detected in 8/188 (4.2%) fecal specimens from healthy children.

### CyCV-VN detection in animal samples.

Previous reports have shown the detection of diverse cycloviruses in farm animal meats (3). The existence of possible animal reservoirs as potential sources for human exposure was investigated using CyCV-VN-specific real-time PCR. We tested fecal material from poultry and pigs from the home province (Dong Thap) of the “index” patient from whom the complete genome of CyCV-VN was obtained. Viral DNA was detected in 38/65 (58%) animal specimens, including 12/20 (60%) pigs (5/10 boot swabs and 7/10 individual fecal droppings), 12/12 (100%) chickens (3/3 boot swabs and 9/9 individual fecal droppings), and 14/33 (42%) ducks (6/13 boot swabs and 8/20 individual fecal droppings).

### Genetic diversity of CyCV-VN.

In order to explore the genetic relationship between the cycloviruses detected in human and animal specimens, we obtained additional 6 full-genome sequences (4 from human CSF and 2 from chicken and pig feces), 1 nearly full-genome sequence from a duck fecal sample, and 19 partial Rep gene sequences (10 from CSF, 4 from human stool samples, and 5 from animal fecal samples). Pairwise comparison of the obtained sequences revealed that the obtained human and animal cyclovirus sequences belong to the same CyCV-VN species, as reflected by a degree of sequence similarity of >97% between the detected strains (data not shown). This low intraspecies sequence divergence of the CyCV-VN is consistent with that of other cyclovirus species (3–5). The results of phylogenetic analyses are presented in Fig. 3.

## DISCUSSION

The availability of highly sensitive molecular methods such as PCR and sequence-independent techniques based on next-generation sequencing technology allows enhanced detection of (novel and unknown) pathogens in clinical specimens, which is essential to guide prevention strategies, aid clinical management, and identify the sources of outbreaks (8). Using a sequence-independent virus discovery assay, VIDISCA-454, we identified a cycloviral sequence in the CSF of patients with acute encephalitis with unknown etiology. Whole-genome characterization indicated that the sequence represented a novel species within the genus *Cyclovirus* of the family *Circoviridae* that we tentatively named CyCV-VN.

*Circoviridae* is a family of nonenveloped viruses containing a circular single-stranded DNA genome and is subdivided into two genera, *Circovirus* and *Gyrovirus*, both of which have been associated only with disease in animals, including birds and pigs. Avian circoviruses have been associated with beak abnormalities, lethargy, and anorexia in pigeons and production losses and mortality in geese (9), and porcine circovirus 2 causes respiratory and enteric diseases, dermatitis, and reproductive problems in pigs, resulting in economic losses in the pork industry (10–12). Recently, novel viruses belonging to the family *Circoviridae* have been identified which have been proposed to represent a novel genus, *Cyclovirus* (3). In addition to being found in farm animals (e.g., sheep and bovines), chimpanzees, bats, cockroaches, and dragonflies (3, 5, 7, 13), cycloviruses have been detected in the blood of patients with febrile illness, stool samples of children with acute flaccid paralysis, and skin swabs and feces of healthy adults and children, respectively (3, 14, 15). However, a causal link between cycloviruses and human or animal disease has not been proven (3).

After initial discovery and characterization, we detected CyCV-VN by PCR in a total of 12 CSFs from 273 patients with acute encephalitis of unknown etiology. We also tested the CSF from patients with other laboratory-confirmed CNS infections and found a detection rate similar to that in patients with unknown etiology (16/369; 4.3%). In contrast, CyCV-VN DNA was not detected in 122 CSF specimens from patients with noninfectious CNS conditions (*P* = 0.015). However, it should be noted that 88% of 122 CSF samples from patients without CNS infections were collected over a short period of time (August to October 2011), possibly introducing bias.

The detection of CyCV-VN in CSF of patients with CNS infections, both with known and with unknown etiology, but not in CSF from patients with noninfectious CNS disorders may imply a pathogenic role of CyCV-VN, either as a single agent or as a coinfecting agent, e.g., by enhancing severity of disease or facilitating entry of other pathogens into the CNS compartment. While the mortality of CyCV-VN-infected patients seemed relatively low, comparison of clinical severities and outcomes between CyCV-VN patients with and without coinfection with other pathogens could not be done reliably because of incomplete data collection at discharge and the small numbers of patients (Table 2).

CyCV-VN was detected in patients of all ages, from less than 1 year to over 60 years, from 7 different provinces in southern and central Vietnam over a 10-year period from 1999 to 2009. This suggests that this virus can affect persons of all ages, has a broad geographic distribution in Vietnam, and did not emerge very recently.

No CyCV-VN DNA was found in 3 available parallel sera of CSF-positive patients, suggesting that the detection of CyCV-VN DNA in CSF does not reflect ongoing viremia with spillover into the CSF or blood contamination of CSF after possible traumatic lumbar puncture. However, the possibility that past viremia was responsible for CNS entry cannot be excluded. The fact that CyCV-VN was not detected in 90 randomly selected blood from Vietnamese subjects renders it unlikely that cyclovirus is a ubiquitous commensal virus, like torque teno virus (TTV), which is asymptomatically present in blood of 7 to 78% of people, with occasional detection in CSF (16–21).

CyCV-VN DNA was also detected in throat and rectal swabs of CSF-positive patients as well as in fecal specimens of healthy children. This suggests a possible food-borne or fecal-oral transmission route for infection, although detection in feces of healthy children may also merely reflect consumption of CyCV-VN-infected food rather than true infection.

To explore the existence of possible animal reservoirs of CyCV-VN for such transmission, we tested fecal samples from animals in Dong Thap Province, where the index patient lived, and found remarkably high detection rates in fecal samples collected from pigs, ducks, and chickens, ranging from 42 to 100%. Sequence identities between CyCV-VN strains in humans and animals were high (around 98%) (Fig. 3), which may suggest interspecies transmission between animals and possibly from animals to humans.

It should be noted that the PCR screening method was designed for specific detection of only CyCV-VN and closely related viruses and that complete or partial genomic sequences were not obtained in all CyCV-VN PCR-positive samples; hence, the possibility exists that genetically diverse, more distantly related CyCV species are cocirculating in Vietnam. Future research is needed to investigate this possibility and to determine whether human infections are restricted to CyCV-VN species. While the discovery of CyCV-VN and its frequent detection in the usually sterile CSF compartment are remarkable in themselves, the results of the present study should be interpreted with caution, because the mere detection of a novel virus in clinical specimens of patients with disease does not prove a causative role (22, 23). This is illustrated by the recent discovery and subsequent “dediscovery” of xenotropic murine leukemia virus-related virus in patients with prostate cancer and patients with chronic fatigue syndrome (24, 25), which attracted much attention among scientists, the media, and patient groups and required stringent follow-up studies to show the absence of a link between the virus and the disease or syndrome (26, 27). According to criteria recently proposed by Lipkin, our findings only point to a possible causal relationship at present (22, 23). In order to detect a more definitive causal link between infection with CyCV-VN and CNS disease, studies to determine whether CyCV-VN fulfills Koch’s postulates or adapted versions of these are necessary (22, 23, 28–30). For these, efforts to isolate the virus in cell culture or animal models and the detection of a specific immune response to CyCV-VN are imperative and ongoing.

## MATERIALS AND METHODS

### Patients and samples.

Clinical data and CSF specimens analyzed in this study were derived from three prospective clinical studies designed to establish the etiology of CNS infections in Vietnam. These included a previously published study (PVE) on children with presumed viral encephalitis admitted to Children’s Hospital Number One in Ho Chi Minh City in 2004 (*n* = 194) (31); a study (BMD) on adults with clinically suspected viral encephalitis admitted to the Hospital for Tropical Diseases, Ho Chi Minh City, between 1997 and 2008 (*n* = 291) (unpublished data), and a study (01SS) of children and adults with suspected CNS infections admitted to 13 provincial hospitals in central and southern Vietnam between 2008 and 2010 (*n* = 1,241) (32).

Acute-infection CSF specimens were collected, divided into aliquots, and stored at −80°C until analysis. In addition, acute-infection blood specimens and rectal and throat swabs were collected from children participating in the PVE study. Detailed demographic and clinical data, including routine blood and CSF hematology and chemistry laboratory investigations, were collected on case record forms at enrollment and during follow-up.

In all three studies, initial diagnostic efforts included CSF Gram stains, CSF culture, and specific PCR-based methods and serology targeting herpes simplex viruses, enteroviruses, JEV, dengue virus, *Streptococcus pneumoniae*, *Streptococcus suis* serotype 2, *Haemophilus influenzae*, and *Neisseria meningitidis*. Additional diagnostic tests for flaviviruses (generic), influenza A virus, and cytomegalovirus were performed in the PVE and BMD studies. Parechoviruses and Semliki Forest virus were also tested for in the PVE study (33). Adult patients in the BMD study were further tested for varicella zoster virus, influenza B virus, Epstein-Barr virus, mumps virus, and Nipah virus by PCR and for *Mycobacterium tuberculosis* by CSF culture and microscopy. A subset of 121 patients in the 01SS cohort with clinically suspected tuberculosis meningitis was also tested by *M. tuberculosis* PCR (32).

From these case series, CSF specimens with unknown etiology were selected for VIDISCA-454 analysis, for which the following selection criteria were used to increase the probability of detecting a novel viral pathogen causing relevant disease: (i) fever or a history of fever (≥37.5°C), (ii) illness onset of ≤5 days, (iii) CSF white cell count between 6 and 1,000 cells/µl, (iv) normal CSF glucose (CSF glucose/blood glucose ratio ≥ 0.5), and (v) absence of an etiological diagnosis by the methods described above. In total, 125 CSF specimens from patients with CNS infections with unknown etiology (PVE, *n* = 6; BMD, *n* = 45; and 01SS, *n* = 74) were selected.

After the discovery of the novel cyclovirus in the initial clinical series, we investigated its clinical relevance and potential animal reservoirs by further testing of CSF specimens from patients with infectious and noninfectious neurological illness, residual blood specimens, stool samples from healthy children, and fecal samples from poultry and pigs in Vietnam (Table 5).

**TABLE 5:** Samples for PCR screening of CyCV-VN

Sample^*a*^	Disease/syndrome	No.	Collection period	Hospital^*b*^	Hospital location
CSF^*c*^	CNS	642	1999-2009	—^*a*^	Southern and central Vietnam
CSF^*d*^	Noninfectious condition	122	1997-2011	Hospital for Tropical Diseases and Cho Ray Hospital	Ho Chi Minh City, southern Vietnam
Blood^*e*^	Unknown	90	2009-2010	Hue Central Hospital	Hue City, central Vietnam
Human feces	Healthy children	188	2011	NA	Southern Vietnam
Animal feces^*f*^	NA	65	2011	NA	Dong Thap Province, southern Vietnam

^a^ Prior to PCR screening, nucleic acid was isolated from clinical samples with use of the easyMAG (bioMérieux, Marcy l’Étoile, France) or the MagNA pure96 system (Roche), following the manufacturer’s instructions.

^b^ The hospitals from which the noninfectious CSF and blood samples were collected are referral hospitals for southern and central provinces in Vietnam. NA, not applicable.

^c^ From children and adult patients enrolled in the BMD, PVE, or 01SS study (see Materials and Methods for study details).

^d^ Indications for lumbar puncture included headache/migraine (*n* = 17), trauma (*n* = 16), postoperative CSF leakage (*n* = 2), tumors (*n* = 13), benign intracranial hypertension (*n* = 1), epilepsy/convulsion (*n* = 15), encephalopathy (*n* = 5), external ventricular draining (*n* = 4), hemorrhage/stroke (*n* = 29), Guillain-Barré syndrome (*n* = 3), cranial nerve palsies (*n* = 6), and others (*n* = 11). The patients included both children and adults [age median (range): 36 (1 to 91) years; 67% male], and they were from 26 provinces in central or southern Vietnam.

^e^ Anonymized residual blood samples from an influenza sero-surveillance study.

^f^ Including samples collected from individual animals and boot swabs—from pigs (*n* = 20), chickens (*n* = 12), and ducks (*n* = 33).

### VIDISCA-454 analysis.

The selected CSF specimens were analyzed by VIDISCA-454 either as single samples (*n* = 27, for samples with CSF cell counts of ≥152 cells/µl) or as pools of ≤5 specimens (*n* = 23, for the remaining 98 samples with CSF cell counts of ≤151 cells/µl).

The VIDISCA-454 procedure was done as previously described (2). In brief, prior to nucleic acid (NA) isolation, samples were centrifuged to eliminate cell debris and mitochondria, followed by DNA digestion by Turbo DNase I (Ambion, Austin, TX). NAs were then extracted by a guanidinium thiocyanate-based procedure (34) and recovered in 50 µl of sterile water containing a 10 µM concentration of rRNA-blocking oligonucleotides (2 µM for each oligonucleotide). Blocking oligonucleotides were used to avoid reverse transcription and amplification of abundant human rRNA, thereby increasing the sensitivity of the VIDISCA-454 (2). The sequences of these oligonucleotides were designed based on human 18S and 28S rRNA sequences but contain a 3′ dideoxy-C-6 modification which cannot be extended. Reverse transcription of viral RNA was done using nonribosomal hexamer primers (35) and Superscript II reverse transcriptase (Invitrogen, Carlsbad, CA), followed by double-stranded-DNA (dsDNA) synthesis using DNA polymerase I Klenow fragment (Westburg, Leusden, the Netherlands) and RNase H (Amersham, Uppsala, Sweden). The resulting dsDNA was subjected to restriction enzyme digestion with MseI (New England Biolabs, Ipswich, MA), MseI-adaptor ligation with DNA ligase (Invitrogen), and PCR amplification with primers containing a restriction site adaptor and a multiplex identifier (MID; Roche Diagnostics GmbH, Mannheim, Germany). Finally, the amplified product was subjected to emulsion PCR (GS emPCR kit II; Roche) followed by DNA sequencing on a FLX genome sequencer (454 Life Science, Roche Applied Science, Roche), following the supplier’s instructions.

### Conventional and real-time PCR.

To confirm the presence of the cyclovirus-like sequence generated by high-throughput sequencing, specific PCR primers (CyCV31-53F and CyCV178-53R) (Table 1) were designed based on the obtained sequence and were used to test the 5 individual original CSF specimens of the initial pool in which the sequence was detected. Sequence alignment indicated that these primers could detect CyCV-VN and a closely related CyCV species—CyCV-20—classified by Li et al. (3) (see Fig. S1 in the supplemental material). The PCR was done in a final 25-µl volume reaction mixture containing 1.25 U of HotStarTaq DNA polymerase (Qiagen GmbH, Hilden, Germany), an 800 µM concentration of each primer, a 400 µM concentration of each deoxynucleoside triphosphate (dNTP), 5 mM MgCl_2_ (provided with the polymerase), 1× PCR buffer (provided with the polymerase), and 3 µl of extracted NAs. PCR was performed in a Mastercycler (Eppendorf, Hamburg, Germany) with an initial polymerase activation step at 95°C for 14 min 30 s, followed by 45 cycles of 95°C for 30 s, 55°C for 30 s, and 72°C for 30 s. Confirmation of amplified product was done by sequencing, using BigDye Terminator v1.1 cycle sequencing kit (Applied Biosystems, Carlsbad, CA) in an ABI377 automatic sequencer (Applied Biosystems), following the manufacturer’s instructions.

For high-throughput screening of clinical specimens, we designed a real-time PCR using the same primers and a 6-carboxyfluorescein (FAM)-labeled probe (CyCV65-96) (Table 1) at a final concentration of 100 µM. The real-time PCR was performed on a Chromo4 platform (MJ Research, Bio-Rad, Hercules, CA) or a LightCycler480 real-time PCR system (Roche) using the above-described thermal cycling program.

### Inverse PCR and (full-length) genome sequencing.

For whole-genome sequencing, iPCR was employed and performed in a final reaction volume of 50 µl containing 400 µM of the primers CyCV31-53F-rc and CyCV178-53R-rc (Table 1), 250 µM of each dNTPs (Roche Diagnostics), 1× PFU ultra buffer (provided with the polymerase), 1 µl of PfuUltra II fusion HS DNA polymerase (Stratagene, Santa Clara, CA), and 5 µl of extracted NAs. PCR was performed in a Mastercycler (Eppendorf, Germany) with an initial polymerase activation step at 95°C for 2 min, followed by 50 cycles of 95°C for 20 s, 55°C for 20 s, and 72°C for 1 min.

To obtain a partial replication associated protein-coding gene sequence, primers CyCV-FL3 and CyCV-FL4 (Table 1) were employed, and the PCR was carried out with PfuUltra II fusion HS DNA polymerase under the conditions used for iPCR.

The product was directly sequenced in duplicate using the same PCR primers and walking primers (CyCV-FL1 to -FL4) (Table 1), whose design was based on the sequences obtained with the iPCR primers. All sequencing reactions were done using a BigDye Terminator v1.1 cycle sequencing kit (Applied Biosystems), following the manufacturer’s instructions.

All the PCR experiments were performed in molecular diagnostic facilities that consist of three physically separated laboratories for reagent preparation, extraction, and amplification, and these used a unidirectional workflow.

### Sequence analysis.

Sequence reads were imported into CodonCode Aligner v. 3.7.1 (CodonCode, Dedham, MA) to remove adaptor sequences and DNA fragments of *Escherichia coli* genome. The remaining reads were then subjected to a sequence similarity search by BLASTn. Pairwise comparisons and phylogenetic analyses were done using AlignX (Vector NTI Advance 11; Invitrogen) and neighbor-joining in MEGA version 4 (36), respectively. Prediction of ORF of the virus genome was done using Vector NTI advance 11 (Invitrogen).

### Ethics statement.

All clinical studies were reviewed and approved by the Institutional Review Boards of collaborating hospitals/authorities and the Oxford Tropical Research Ethics Committee (OxTREC), University of Oxford, United Kingdom.

### Nucleotide sequence accession number.

The GenBank accession numbers for the CyCV-VN sequences are KF031465–KF031492.

## SUPPLEMENTAL MATERIAL

Figure S1 Nucleotide sequence alignment showing sequence identity between primers/probe of CyCV-VN PCR used and CyCV-VN and CyCV-20 (including CyCV-TN8, -TN15, -TN16, -TN18, -TN22, and -TN25) sequences. Degenerate nucleotides: D = A, G, or T; M = A or C; S = G or C. Download Figure S1, TIF file, 1.2 MB

## References

[1] Granerod J Tam CC Crowcroft NS Davies NW Borchert M Thomas SL 2010 Challenge of the unknown. A systematic review of acute encephalitis in non-outbreak situations. Neurology 75:924–932. 20820004 10.1212/WNL.0b013e3181f11d65

[2] de Vries M Deijs M Canuti M van Schaik BD Faria NR van de Garde MD Jachimowski LC Jebbink MF Jakobs M Luyf AC Coenjaerts FE Claas EC Molenkamp R Koekkoek SM Lammens C Leus F Goossens H Ieven M Baas F van der Hoek L 2011 A sensitive assay for virus discovery in respiratory clinical samples. PLoS One 6:e16118 http://dx.doi.org/10.1371/journal.pone.0016118. 21283679 10.1371/journal.pone.0016118 PMC3025933

[3] Li L Kapoor A Slikas B Bamidele OS Wang C Shaukat S Masroor MA Wilson ML Ndjango JB Peeters M Gross-Camp ND Muller MN Hahn BH Wolfe ND Triki H Bartkus J Zaidi SZ Delwart E 2010 Multiple diverse circoviruses infect farm animals and are commonly found in human and chimpanzee feces. J. Virol. 84:1674–1682. 20007276 10.1128/JVI.02109-09 PMC2812408

[4] Li L Shan T Soji OB Alam MM Kunz TH Zaidi SZ Delwart E 2011 Possible cross-species transmission of circoviruses and cycloviruses in farm animals. J. Gen. Virol. 92:768–772. 21177928 10.1099/vir.0.028704-0 PMC3133700

[5] Rosario K Marinov M Stainton D Kraberger S Wiltshire EJ Collings DA Walters M Martin DP Breitbart M Varsani A 2011 Dragonfly cyclovirus, a novel single-stranded DNA virus discovered in dragonflies (*Odonata*: *Anisoptera*). J. Gen. Virol. 92:1302–1308. 21367985 10.1099/vir.0.030338-0

[6] Todd D 2005 Circoviridae, p 326–334. *In* Fauquet CM Mayo MA Maniloff J Desselberger U Ball LA, Virus taxonomy. Eighth Report of the International Committee on Taxonomy of Viruses. Elsevier Academic, San Diego, CA.

[7] Padilla-Rodriguez M Rosario K Breitbart M Novel cyclovirus discovered in the Florida woods cockroach *Eurycotis floridana* (Walker). Arch. Virol., 2013 Jan 29. [Epub ahead of print] . 10.1007/s00705-013-1606-x 23358613

[8] Morse SS Mazet JA Woolhouse M Parrish CR Carroll D Karesh WB Zambrana-Torrelio C Lipkin WI Daszak P 2012 Prediction and prevention of the next pandemic zoonosis. Lancet 380:1956–1965. 23200504 10.1016/S0140-6736(12)61684-5 PMC3712877

[9] Todd D 2004 Avian circovirus diseases: lessons for the study of PMWS. Vet. Microbiol. 98:169–174. 14741130 10.1016/j.vetmic.2003.10.010

[10] Allan GM Ellis JA 2000 Porcine circoviruses: a review. J. Vet. Diagn. Invest. 12:3–14. 10690769 10.1177/104063870001200102

[11] Opriessnig T Meng XJ Halbur PG 2007 Porcine circovirus type 2 associated disease: update on current terminology, clinical manifestations, pathogenesis, diagnosis, and intervention strategies. J. Vet. Diagn. Invest. 19:591–615. 17998548 10.1177/104063870701900601

[12] Ramamoorthy S Meng XJ 2009 Porcine circoviruses: a minuscule yet mammoth paradox. Anim. Health Res. Rev. 10:1–20. 18761774 10.1017/S1466252308001461

[13] Li L Victoria JG Wang C Jones M Fellers GM Kunz TH Delwart E 2010 Bat guano virome: predominance of dietary viruses from insects and plants plus novel mammalian viruses. J. Virol. 84:6955–6965. 20463061 10.1128/JVI.00501-10 PMC2898246

[14] Yozwiak NL Skewes-Cox P Stenglein MD Balmaseda A Harris E DeRisi JL 2012 Virus identification in unknown tropical febrile illness cases using deep sequencing. PLoS Negl. Trop. Dis. 6:e1485 http://dx.doi.org/10.1371/journal.pntd.0001485. 22347512 10.1371/journal.pntd.0001485 PMC3274504

[15] Foulongne V Sauvage V Hebert C Dereure O Cheval J Gouilh MA Pariente K Segondy M Burguière A Manuguerra JC Caro V Eloit M 2012 Human skin microbiota: high diversity of DNA viruses identified on the human skin by high throughput sequencing. PLoS One 7:e38499 http://dx.doi.org/10.1371/journal.pone.0038499. 22723863 10.1371/journal.pone.0038499 PMC3378559

[16] Maggi F Fornai C Vatteroni ML Siciliano G Menichetti F Tascini C Specter S Pistello M Bendinelli M 2001 Low prevalence of TT virus in the cerebrospinal fluid of viremic patients with central nervous system disorders. J. Med. Virol. 65:418–422. 11536254 10.1002/jmv.2051

[17] Urwijitaroon Y Barusrux S Chunlertlith K Mairiang P Yoshimura H 2007 Torquetenovirus infection among Northeastern Thai blood donors. Southeast Asian J. Trop. Med. Public Health 38:686–689. 17883007

[18] de Castro Amarante MF Kashima S Covas DT 2007 TT virus (TTV) genotyping in blood donors and multiple transfused patients in Brazil. Virus Genes 35:503–509. 17570047 10.1007/s11262-007-0124-x

[19] Ataei B Emami Naeini A Khorvash F Yazdani MR Javadi AA 2012 Prevalence of transfusion transmitted virus infection in hemodialysis patients and injection drug users compared to healthy blood donors in Isfahan, Iran. Gastroenterol. Res. Pract. 2012:671927 http://dx.doi.org/10.1155/2012/671927. 10.1155/2012/671927 PMC3507261 23213328

[20] Hashish MH El-Barrawy MA Mahmoud OA Abdel Rahman NW 2005 TT virus among blood donors in Alexandria. J. Egypt. Public Health Assoc. 80:651–664. 17187747

[21] Grabarczyk P Brojer E Windyga J Łopaciuk S Klukowska A Mikulska M 2006 GBV-C/HGV and TTV infection markers in Polish blood donors and haemophilia patients. Przegl. Epidemiol. 60:581–588. 17249183

[22] Lipkin WI 2013 The changing face of pathogen discovery and surveillance. Nat. Rev. Microbiol. 11:133–141. 23268232 10.1038/nrmicro2949 PMC4098826

[23] Lipkin WI 2010 Microbe hunting. Microbiol. Mol. Biol. Rev. 74:363–377. 20805403 10.1128/MMBR.00007-10 PMC2937520

[24] Lombardi VC Ruscetti FW Das Gupta J Pfost MA Hagen KS Peterson DL Ruscetti SK Bagni RK Petrow-Sadowski C Gold B Dean M Silverman RH Mikovits JA 2009 Detection of an infectious retrovirus, XMRV, in blood cells of patients with chronic fatigue syndrome. Science 326:585–589. 19815723 10.1126/science.1179052

[25] Urisman A Molinaro RJ Fischer N Plummer SJ Casey G Klein EA Malathi K Magi-Galluzzi C Tubbs RR Ganem D Silverman RH DeRisi JL 2006 Identification of a novel gammaretrovirus in prostate tumors of patients homozygous for R462Q RNASEL variant. PLoS Pathog. 2:e25 http://dx.doi.org/10.1371/journal.ppat.0020025. 16609730 10.1371/journal.ppat.0020025 PMC1434790

[26] Alter HJ Mikovits JA Switzer WM Ruscetti FW Lo SC Klimas N Komaroff AL Montoya JG Bateman L Levine S Peterson D Levin B Hanson MR Genfi A Bhat M Zheng H Wang R Li B Hung GC Lee LL Sameroff S Heneine W Coffin J Hornig M Lipkin WI 2012 A multicenter blinded analysis indicates no association between chronic fatigue syndrome/myalgic encephalomyelitis and either xenotropic murine leukemia virus-related virus or polytropic murine leukemia virus. mBio 3:e00266-12 http://dx.doi.org/10.1128/mBio.00266-12. 22991430 10.1128/mBio.00266-12 PMC3448165

[27] Lee D Das Gupta J Gaughan C Steffen I Tang N Luk KC Qiu X Urisman A Fischer N Molinaro R Broz M Schochetman G Klein EA Ganem D Derisi JL Simmons G Hackett J Silverman RH Chiu CY 2012 In-depth investigation of archival and prospectively collected samples reveals no evidence for XMRV infection in prostate cancer. PLoS One 7:e44954 http://dx.doi.org/10.1371/journal.pone.0044954. 23028701 10.1371/journal.pone.0044954 PMC3445615

[28] Fredericks DN Relman DA 1996 Sequence-based identification of microbial pathogens: a reconsideration of Koch’s postulates. Clin. Microbiol. Rev. 9:18–33. 8665474 10.1128/cmr.9.1.18 PMC172879

[29] Mokili JL Rohwer F Dutilh BE 2012 Metagenomics and future perspectives in virus discovery. Curr. Opin. Virol. 2:63–77. 22440968 10.1016/j.coviro.2011.12.004 PMC7102772

[30] Rivers TM 1937 Viruses and Koch’s postulates. J. Bacteriol. 33:1–12. 16559982 10.1128/jb.33.1.1-12.1937 PMC545348

[31] Le VT Phan TQ Do QH Nguyen BH Lam QB Bach V Truong H Tran TH Nguyen V Tran T Vo M Tran VT Schultsz C Farrar J van Doorn HR de Jong MD 2010 Viral etiology of encephalitis in children in southern Vietnam: results of a one-year prospective descriptive study. PLoS Negl. Trop. Dis. 4:e854 http://dx.doi.org/10.1371/journal.pntd.0000854. 21049060 10.1371/journal.pntd.0000854 PMC2964288

[32] Ho Dang Trung N Le Thi Phuong T Wolbers M Nguyen Van Minh H Nguyen Thanh V Van MP Thieu NT Van TL Song DT Thi PL Thi Phuong TN Van CB Tang V Ngoc Anh TH Nguyen D Trung TP Thi Nam LN Kiem HT Thi Thanh TN Campbell J Caws M Day J de Jong MD Van Vinh CN Van Doorn HR Tinh HT Farrar J Schultsz C VIZIONS CNS Infection Network 2012 Aetiologies of central nervous system infection in Viet Nam: a prospective provincial hospital-based descriptive surveillance study. PLoS One 7:e37825 http://dx.doi.org/10.1371/journal.pone.0037825. 22662232 10.1371/journal.pone.0037825 PMC3360608

[33] Tan LV Ha DQ Hien VM van der Hoek L Farrar J de Jong MD 2008 Me Tri virus: a Semliki Forest virus strain from Vietnam? J. Gen. Virol. 89:2132–2135. 18753222 10.1099/vir.0.2008/002121-0

[34] Boom R Sol CJ Salimans MM Jansen CL Wertheim-van Dillen PM van der Noordaa J 1990 Rapid and simple method for purification of nucleic acids. J. Clin. Microbiol. 28:495–503. 1691208 10.1128/jcm.28.3.495-503.1990 PMC269651

[35] Endoh D Mizutani T Kirisawa R Maki Y Saito H Kon Y Morikawa S Hayashi M 2005 Species-independent detection of RNA virus by representational difference analysis using non-ribosomal hexanucleotides for reverse transcription. Nucleic Acids Res. 33:e65. 15817564 10.1093/nar/gni064 PMC1074749

[36] Tamura K Dudley J Nei M Kumar S 2007 MEGA4: molecular evolutionary genetics analysis (MEGA) software version 4.0. Mol. Biol. Evol. 24:1596–1599. 17488738 10.1093/molbev/msm092

