# Supplementary figures and images for: Identification of a New Cyclovirus in Cerebrospinal Fluid of Patients with Acute Central Nervous System Infections
Source: mBio. 2013 Jun 18;4(3):e00231-13. doi: 10.1128/mBio.00231-13 (PMC3684831; doi:10.1128/mBio.00231-13)

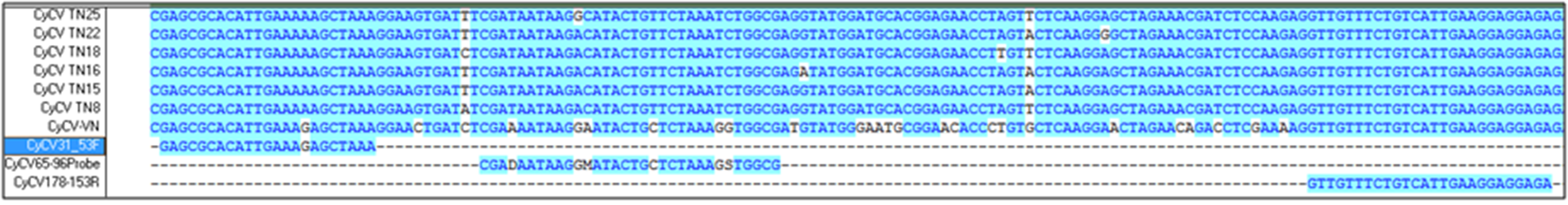

Supplement: Figure S1 — Nucleotide sequence alignment showing sequence identity between primers/probe of CyCV-VN PCR used and CyCV-VN and CyCV-20 (including CyCV-TN8, -TN15, -TN16, -TN18, -TN22, and -TN25) sequences. Degenerate nucleotides: D = A, G, or T; M = A or C; S = G or C. Download [file mbo003131535sf01.tif]
